# Estimating the economic and social consequences for patients diagnosed with human African trypanosomiasis in Muchinga, Lusaka and Eastern Provinces of Zambia (2004–2014)

**DOI:** 10.1186/s40249-017-0363-6

**Published:** 2017-10-10

**Authors:** Allan Mayaba Mwiinde, Martin Simuunza, Boniface Namangala, Chitalu Miriam Chama-Chiliba, Noreen Machila, Neil Anderson, Alexandra Shaw, Susan C. Welburn

**Affiliations:** 1 School of Veterinary Medicine, University of Zambia, Lusaka, Zambia; 2 Department of Economics, University of Zambia, Lusaka, Zambia; 3 Division of Infection and Pathway Medicine and Centre for Infectious Diseases, School of Biomedical Sciences, College of Medicine and Veterinary Medicine, The University of Edinburgh, Chancellor’s Building, 49 Little France Crescent, Edinburgh, Scotland EH16 4SB UK; 4 The Royal (Dick) School of Veterinary Studies and the Roslin Institute, University of Edinburgh, Roslin, EH25 9RG UK; 5 AP Consultants, Walworth Enterprise Centre, Andover, SP10 5AP UK; 6 School of Veterinary Medicine Department of Disease Control, University of Zambia, P.O Box 32379, Lusaka, Zambia

**Keywords:** Human African trypanosomiasis, Hat, Sleeping sickness, *T. B. Rhodesiense*, Social and economic burden, DALYs, Zambia

## Abstract

**Background:**

Acute human African trypanosomiasis (rHAT) caused by *Trypanosoma brucei rhodesiense* is associated with high mortality and is fatal if left untreated. Only a few studies have examined the psychological, social and economic impacts of rHAT. In this study, mixed qualitative and quantitative research methods were used to evaluate the socio-economic impacts of rHAT in Mambwe, Rufunsa, Mpika and Chama Districts of Zambia.

**Methods:**

Individuals diagnosed with rHAT from 2004 to 2014 were traced using hospital records and discussions with communities. Either they, or their families, were interviewed using a structured questionnaire and focus group discussions were conducted with affected communities. The burden of the disease was investigated using disability adjusted life years (DALYs), with and without discounting and age-weighting. The impact of long-term disabilities on the rHAT burden was also investigated.

**Results:**

Sixty four cases were identified in the study. The majority were identified in second stage, and the mortality rate was high (12.5%). The total number of DALYs was 285 without discounting or age-weighting. When long-term disabilities were included this estimate increased by 50% to 462. The proportion of years lived with disability (YLD) increased from 6.4% to 37% of the undiscounted and un-age-weighted DALY total. When a more active surveillance method was applied in 2013–2014 the cases identified increased dramatically, suggesting a high level of under-reporting. Similarly, the proportion of females increased substantially, indicating that passive surveillance may be especially failing this group.

An average of 4.9 months of productive time was lost per patient as a consequence of infection. The health consequences included pain, amnesia and physical disability. The social consequences included stigma, dropping out of education, loss of friends and self-esteem. Results obtained from focus group discussions revealed misconceptions among community members which could be attributed to lack of knowledge about rHAT.

**Conclusions:**

The social and economic impact of rHAT on rural households and communities is substantial. Improved surveillance and strengthening of local medical services are needed for early and accurate diagnosis. Disease prevention should be prioritised in communities at risk of rHAT, and interventions put in place to prevent zoonotic disease spill over from domestic animals and wildlife. Supportive measures to mitigate the long-term effects of disability due to rHAT are needed.

**Electronic supplementary material:**

The online version of this article (10.1186/s40249-017-0363-6) contains supplementary material, which is available to authorized users.

## Multilingual abstract

Please see Additional file 1 for translation of the abstract into the five official working languages of the United Nations.

## Background

Human African trypanosomiasis (HAT), or sleeping sickness, is a vector-borne neglected tropical disease (NTD) caused by two morphologically indistinguishable trypanosome species, *Trypanosoma brucei gambiense* (western and central Africa) and *Trypanosoma brucei rhodosiense* (eastern and southern Africa), spread by tsetse flies (*Glossina ssp*) [1]. *Trypanosoma brucei gambiense* is transmitted through human-tsetse contact and is responsible for the majority of the reported cases [2] while *T. b. rhodesiense* is a complex zoonotic disease involving a wide range of wildlife and livestock reservoirs [1, 3]. Although the two sub-species of *Trypanosoma brucei* are morphologically indistinguishable, they can be differentiated by molecular methods [4]. HAT is invariably fatal if left untreated with death occurring between six to eight months in the case of the acute Rhodesian form (rHAT) or after one or more years in the case of the chronic Gambian form (gHAT) [5].

The early febrile stage for both diseases includes a variety of non-specific sequelae (headache, fever, joint pains, chancre, skin lesion, pruritus and a variety of endocrine, cardiac and gastrointestinal disorders), while the late neurological stage is characterised by walking difficulties, sensory disorders, tremors, sleeping disturbances, coma and other related symptoms [6, 7]. Drug therapies for both early and late stage rHAT have severe and debilitating adverse side-effects; in particular, administration of the arsenic-based drug melarsoprol in late stage rHAT causes encephalopathy in about 5 to 10% of the patients [6].

Approximately 70 million people are at varying degrees of risk from HAT in sub-Saharan Africa with an estimated 21 million living in areas with a moderate to very high risk of infection [8]. HAT has been a major impediment to social and economic development in most sub-Saharan African rural communities, mainly affecting the poorest people in remote rural areas. Based on the disability-adjusted -life year, or DALY (an estimate of the number of years of life lost due to premature death and disability), HAT is estimated to cause approximately 1.6 million DALYs, and is considered second among all vector-borne diseases in Africa for mortality and fourth for related disability [9]. While the DALY has become a ubiquitous metric in global health, important limitations exist in relation to the DALY and HAT [6, 10]. These include the specific disability weights given, accounting for undetected cases and the exclusion of long-term impacts on growth and neurological impairment, either by infection or drug toxicity. While over 175,000 cases of HAT were reported between 2000 and 2009 [11], the disease is found largely in remote rural areas with limited diagnostic capacity and poor access to health facilities [12, 13]. According to the World Health Organization (WHO), in 2009 about half of cases were thought to be unreported [14]. Due to sustained control activities (in particular active case-finding for gHAT) this represented a significant improvement on the situation in the late 1990s when only just over 10% of all HAT cases were reported [14]. In 2009 the total number of reported cases fell below 10,000 for the first time in five decades, by 2015 fewer than 3000 cases were reported, of which 97% were gHAT. However, for rHAT there has been limited active case-finding, so that it is difficult to gauge the level of under-reporting.

The few available district-level DALY studies for HAT have confirmed its significant public health burden in endemic communities [15, 16]. Costs incurred by HAT patients have been calculated in Uganda, Tanzania and the Democratic Republic of Congo (DRC), showing that the disease causes a significant impact on households and health systems [15–17]. Patient expenses at a HAT treatment centre in Tanzania were estimated at US$ 68.40 for indirect medical costs (transport and living expenses) and US$ 25.50 for direct medical costs while costs to the local health services were US$ 83.80 per patient [15]. In the DRC, total treatment costs for gHAT were equivalent to five months of a household’s income, rising to as much as 17 months in cases of disease complications [18]. Health seeking behaviour studies have shown that HAT patients visit numerous public and private health providers over several months before being correctly diagnosed and treated [13, 19, 20]. Recovery periods typically take many months and HAT is thought to lead to many long-term disabilities caused by drug toxicity and infection, although the associated morbidity has not been quantified [21]. The DALY is not the only methodology that can be applied to quantify disease burden; economic calculations are also regularly used at both societal and household level – for example, impact of treatment costs and losses in income to patient households [18].

For many of the NTDs, the burden of disease can also be approached from the perspective of the individual patient where infection has significant social, psychological and livelihood impacts [22–25]. HAT patients mostly live in poor rural settings and are dependent on subsistence agriculture, wildlife ecosystem services and livestock keeping, in the vicinity of tsetse-breeding sites [15–18]. NTDs such as gHAT and rHAT are considered major obstacles to socio-economic development, creating a ‘cycle of poverty’ among affected households, but few disease burden studies have captured the multiple ways in which the NTDs affect household productivity, quality of life and livelihood strategies. Of note, within the community and family structures, rHAT impacts on lives and livelihoods [26]. In Zambia, although rHAT is reported to be in decline [27], sporadic cases of the disease continue to be reported [28].

While the HAT burden may not appear significant in comparison to diseases like HIV/AIDS, tuberculosis or malaria, it is highly focalised and its impact on communities should be assessed at a local-level. Estimating the economic and social consequences of the disease at household level is the best way to accurately ascertain the impact of the disease, focusing on psychological, social and economic impacts for the patient, their household and their community.

This study aimed to assess the economic and social consequences of HAT at household level in four districts where rHAT is endemic; tracing the experience of the household from infection through to treatment pathways, coping strategies, livelihood impacts, the process of diagnosis, rHAT treatment, recovery or disability and death. This information will assist in the broader policy-making and prioritisation of interventions necessary to ease the burden of rHAT in affected areas.

## Methods

### Description of the study area

**Fig. 1:**
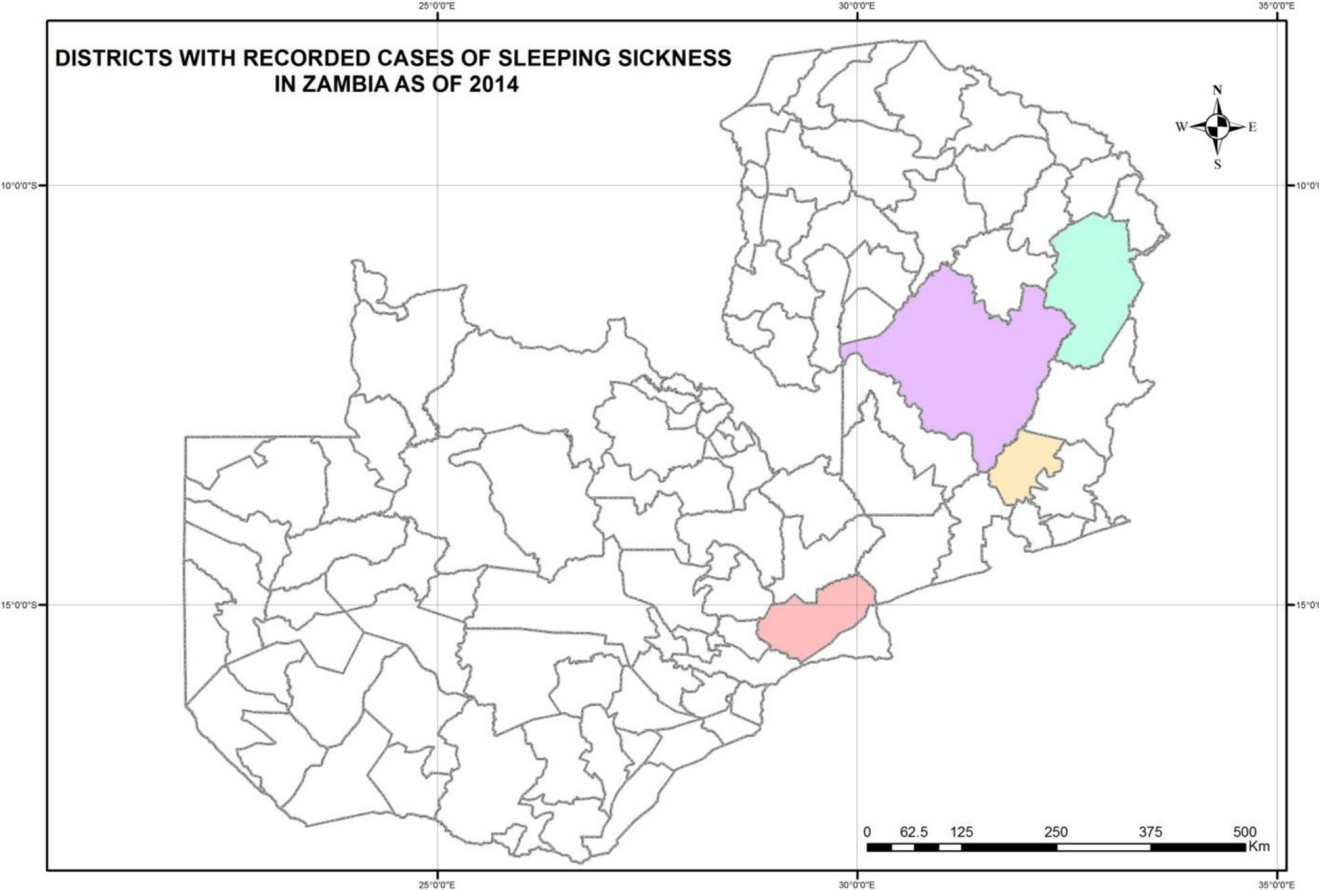
Districts with recorded cases of Sleeping Sickness in Zambia, as of 2014. Red = Rufunsa District, orange = Mambwe District, purple = Mpika District, and blue = Chama District

Data for this study were collected between September and November, 2014. The study area comprised of four districts within Zambia (Rufunsa District in Lusaka Province, Mpika and Chama Districts in Muchinga Province, and Mambwe District in Eastern Province) where sporadic cases of rHAT are reported (Fig. 1) [29]. These areas are predominately rural, where the economy is based on mixed crop-livestock farming and small-scale trades. Over 60% of the population is considered to live below the poverty line, of whom 40% live in absolute poverty. Farming activities revolve around a variety of different main crops such as maize, groundnuts, soya beans, cotton and tobacco, grown during the rainy season. Wetlands are significant areas for keeping cattle (herd sizes are in most cases fewer than 80 cattle), while also facilitating the survival of a low to moderate tsetse population density. The major ethnic groups include Bemba and Bisa in Muchinga Province, Chewa, Nsenga, Tumbuka and Ngoni, in Eastern Province, and Soli in Lusaka Province.

### Study design

Both cross-sectional and retrospective surveys were undertaken to determine the economic and social consequences of rHAT in previously and currently affected rHAT patients, with additional focus group discussions. All patients diagnosed with rHAT from 2004 to 2014 in Zambia’s Lusaka, Eastern and Muchinga Provinces were included in the study. Patients diagnosed with HAT between 2004 and 2012 were identified using passive surveillance methods using hospital records. In 2013 and 2014 this method was supplemented by a degree of active surveillance using discussions with local communities to identify additional patients. Diagnosis was confirmed using polymerise chain reaction (PCR) and/or loop mediated isothermal amplification (LAMP) [27]. In total there were 64 diagnosed HAT cases. Of these, 22 patients came from Chama, 11 from Rufunsa, 28 from Mpika and three from Mambwe Districts. All cases (*n* = 64) were traced and all patients or their families were interviewed with at most two visits.

Structured questionnaires were administered to all patients or their close relatives (care givers) to collect information on the economic and social impacts of rHAT in the communities or districts. Hospital records of patients who were interviewed were retrieved, where possible, to confirm the time period the patient was undergoing treatment. Patient data were obtained together with national statistics from the Ministry of Health.

Focus group discussions were also conducted with the affected communities [30]. The focus groups comprised seven to ten people per discussion and included health workers, people who had suffered from rHAT and their relatives or friends, so as to obtain in-depth information on concepts, perceptions and ideas of the group regarding the social consequences of the disease. A total of eight focus group discussions were conducted during the study, covering all four districts (two in Chama, one in Mambwe, two in Mpika and three in Rufunsa).

### Data analysis

Focus group discussion data were analysed using inductive approaches and thematic coding, carried out by two independent researchers. All structured interview data were entered and analysed using Excel (Microsoft Office Excel® 2007). Qualitative data were entered into Microsoft Word™ and analysed manually based on accepted methods of coding and memo-writing [30, 31]. All transcripts were de-identified and personal identifiers were removed to protect individual patient data. For quantitative data, descriptive statistics were generated for variables under study, and analysis of variance (ANOVA) was used to determine associations among the variables. Significance was accepted at 95% confidence level for all analyses.

### Calculation of DALYs

Disability-adjusted life years were calculated using the DALY calculation template available through the National Tools of the WHO’s Global Burden of Disease website [32]. The template was adapted so that there was one row per patient in the study, enabling each patient to be assigned their own disability weight and duration. Therefore, although in this paper summary figures are provided by age category for convenience, DALYs were actually calculated at the individual level. For years of life lost (YLL), the Population and Deaths per 1000 columns were left blank. The recorded age at death was entered into the respective column for each individual as was the estimated life expectancy at that age (estimated for each individual using the 2010 Census of Population and Housing figures for rural life expectancy [33]).

To calculate the years lived with disability (YLD), the Population column was once again left blank. As each row corresponded to an individual, an incidence of 1 was entered into the Incidence column and the Incidence per 1000 column left blank. For patients who survived infection with rHAT, the ages at onset were calculated by subtracting the time elapsing since diagnosis from the age at interview. For patients who died, the age at onset was calculated by subtracting the time elapsing between diagnosis and death from their age at death. Duration was based on careful questioning of the patients as to how long they had been unable to do their normal activities. This was estimated from the time they first experienced symptoms to the time they resumed productive work. As some patients were still hospitalised or had not started their recovery at the time of the interviews, duration could not be estimated for every patient. For six patients the duration could not be estimated and the average duration for the whole cohort was used instead.

Disability weights of 0.21 and 0.81 were applied for the duration of illness as described above for first and second stage rHAT respectively, as described by Fèvre et al. [16]. Notably, the use of these disability weights in the DALY calculation has the limitation in that it assigns the same disease weighting to individuals of the same stage of the disease, but with different severity. These disability weightings also do not account for long-term sequelae from rHAT, such as disfigurement, physical disability and mental impairment. To investigate the influence of these important consequences of rHAT infection (and treatment) on the DALY estimates, a separate calculation was conducted using additional disability weights for patients recorded as suffering from these disabilities. Disability weights have recently been published in the Global Burden of Disease Study 2015 for patients suffering from the long-term effects of trypanosomiasis [34]. Disability weights of 0.067 for disfigurement due to HAT, and 0.542 for severe motor or cognitive impairment due to HAT, were recommended. To apply these weights in the current study, an additional row was created in the template spreadsheet for all patients who had not fully recovered. A disability weight of 0.067 was applied to all patients who recovered with a minor disability and a weight of 0.542 was applied to those who recovered with a major physical or mental disability (note that this was in addition to the standard disability weights described above). The date of onset was taken as being the date they were no longer absent from work (i.e. when the duration for the acute illness used in the standard DALY calculation ended) and the condition was assumed to be lifelong.

Alongside the standard DALY calculations, DALYs were also calculated with the application of discounting (using a standard rate of 0.03), age-weighting (K = 1 on the WHO template) and both discounting and age-weighting [35]. Discounting aligns with empirical economic evidence [36, 37], in assigning a higher weight to years lost now or in the near future, as compared to years lost in the more distant future [37]). The non-discounted, non-age-weighted DALY estimate is non-discriminatory for age and was adopted as the baseline method for calculation of DALYs for males and females [38].

### Income

A comprehensive estimate of total household income including subsistence farming was outside the scope of this study. Instead, the focus was on estimating household resources for spending on health care, by getting information on cash income, loans and extra sales of agricultural produce used to finance the costs of treatment for rHAT. The currency used during the study period was the United States Dollar (US$), with an exchange rate of 6.3 Zambian Kwacha (ZMW) to one US$.

## Results

### Descriptive statistics

**Fig. 2:**
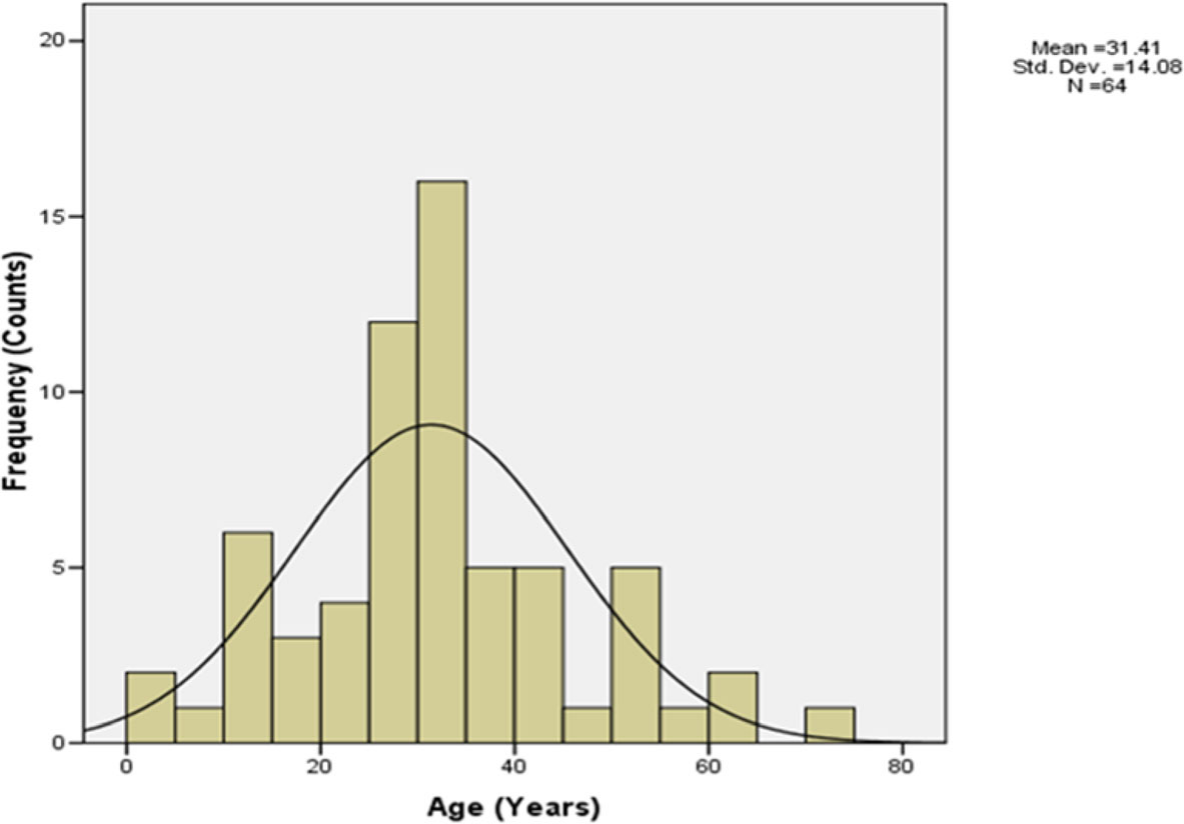
Age distribution of patients included in the study. Mean age = 31.4 years, *SD* = 14.08

Of the 64 patients that were included in the study, there were significantly (*P* < 0.001) more males 45 (70%) than females 19 (30%). The age distribution of the patients ranged from 1 to 72 years, with a mean of 29 years (Fig. 2). Most rHAT patients, 46 (72%) were married, 15 (23%) were single, while only 3 % were widowed. More than half of the patients, 35 (55%) were peasant farmers who grew maize, groundnuts and tobacco.

Health-seeking behaviour was relatively poor among the rHAT patients (Table 1). Thirty one (48%) of the patients sought medical care from a health facility when they first developed signs and symptoms of rHAT. However, 17 (26%) did not seek medical care when they had developed symptoms of rHAT and did not associate the intermittent headache initially experienced as a serious health problem. One patient from Mambwe District believed they had contracted rHAT in 2011 and sought care for more than two years from Kamoto Mission Hospital. The patient was referred to Chipata General Hospital, where rHAT was consistently misdiagnosed, until second stage rHAT was diagnosed in 2014. At this stage the patient was exhibiting impaired judgement and was experiencing sleeping disorders and intermittent headaches. In addition, patients with delayed rHAT diagnosis felt that going to the health centre would be a waste of time and had hoped to recover with self-medication and home-based care as evidenced by the extract from one patient in Mambwe District:

“*I think I have not gotten any help needed in all the hospitals I have attended. In my opinion they don’t know what I am sick of. For this reason, I have decided not to continue seeking medication, but to stay with my disabilities of sleeping disorders, than going back to the hospital where they will just watch me and later tell me to go back home*.” Patient, Mambwe District.

Six (9%) of the patients in this study reported to have sought help from traditional healers before seeking modern medical attention (including one patient who also self-medicated). In some areas, there were suspicions from family, relatives, neighbours and friends that these rHAT patients had been bewitched. The evidence from the focus group discussions established that going to the traditional healers for medication was culturally important and difficult to neglect in rural settings that promote the values of traditional healing. However, seeking treatment from traditional healers incurred high indirect costs at household level. Family members routinely sacrificed animals or agricultural products as a coping mechanism to meet the costs associated with seeking medical assistance from traditional healers. On average, we estimated that an individual would spend $63.49 for the medical services of traditional healers. The cost estimate was obtained by using the approximate market value of the in-kind payments that patients indicated as having paid for the services. This was because none of the patients who sought interventions from traditional healers could recall the exact total costs of items given in exchange for the traditional medicines, partly due to lapse of time. It was mentioned in a focus group discussion that traditional healers were perceived as taking economic advantage of their clients; consulting, diagnosing, prescribing and searching for traditional herbs were considered expensive constraints. When money was unavailable, payments in kind of livestock (chickens, goats, pigs and cattle) were offered cover these costs.

**Table 1:** Health seeking behaviour of HAT patients when they first felt ill

Action by patient	Number of patients	Percent
Went to the clinic	31	48
Self-medication	2	3
Went to traditional healer	5	8
Did nothing	17	26
No action and clinic visit	2	3
Self-medication and clinic visit	5	8
Healer and self-medication	1	2
No reply	1	2
Total	64	100

At least six (9%) rHAT patients self-medicated prior to seeking treatment at the health centres, resulting in delays in cases seeking proper medical care and a late rHAT diagnosis. The average estimated costs of self-medication were US$ 98.10 per patient.

### Household cash income earned by rHAT patients

The gross cash income earned by rHAT patient households when in normal health was determined (Table 2). The median income earned was highest in Chama District (US$ 63.2), while patients from Mpika earned the lowest (US$ 15.6). There was significant difference in the mean income of rHAT patients among the districts (*P* < 0.05), with patients from Chama earning significantly more income than those from Mpika (*P* = 0.019).

**Table 2:** Patients’ monthly household gross cash income by district

District	Number of patients	Median US$	Inter-quartile range US$	Minimum US$	Maximum US $
Chama^a^	22	63.2	31.8–174.6	10.3	1111.1
Mambwe^b^	3	31.8	–	31.8	39.7
Mpika	28	15.6	9.9–17.5	4.8	79.4
Rufunsa^c^	10	39.7	15.9–17.4	9.5	317.5
Total	63	63.2	15.4–63.5	4.8	1111.1

^a^ One household had a monthly income of $ 1111.11, excluding this outlier would have reduced the average income for Chama to $161.7 and for the whole dataset to $61.2. For the whole dataset, 10 households had incomes over $100, of these 5 were over $300 and 8 were in Chama District and 2 in Rufunsa, thus none in Mambwe or Mpika

^b^ No interquartile range is presented for Mambwe as there were only 3 patients

^c^ Household cash income could not be obtained for one patient

### Indirect costs borne by patients

The indirect costs faced by rHAT patients were considerable (Table 3). These include transport, meals, washing soap, additional food and other materials required by the care-giver and patient. In Mpika District, indirect costs were lower because patients had to walk from their rural health centre to Chilonga Mission Hospital. The average cost of transport was calculated for all the four districts combined. The average amount of money that interviewees reported spending on patient’s transport to and from a health centre was US$ 99.3, averaged over all the districts. The amount spent on meals varied depending on the number of days the patient stayed in the hospital. The average amount was estimated at US$ 66.5 per month (in addition to food provided by the hospital which in most cases was not sufficient). In addition, an average of US$ 21.4 was spent on other expenditure such as washing paste, washing soap, toilet paper, bathing basins and additional food.

**Table 3:** Patients’ costs incurred during hospitalisation

Type of expenditure	Total spent by whole patient cohort (US$)	Number of respondents spending on that category	Percentage of the whole patient cohort	Average spent per patient (US$)^a^
Transport	2980.1	30	47	99.3
Medication	504.1	6	9	84.0
Meals	1729.3	26	41	66.5
Washing paste	80.3	12	19	6.7
Food	117.6	8	13	14.7
Total	5411.3	33	52	164.0

^a^ Average of those patients who spent money on this category, not of the whole patient cohort

### Treatment costs

The patients who self-medicated, either sought treatment from traditional healers or were misdiagnosed at health centres, incurred substantial treatment costs averaging US$ 71.9 before being definitively diagnosed. Patients living in rural areas sold agricultural products such as livestock and cereals to sell and cater for medication (11 households, average amount US$ 302.2).

### Period of lost income and household coping mechanisms

The monthly cash income per rHAT patient household ranged from US$ 4.8 to US$ 1111.1 (Table 2). The lowest number of months an individual would lose if diagnosed with rHAT was one month and the highest 36 months (average 4.9 months). Of the 33 households who spent money, the average spend was 4.5 months of household cash income. Within this, there was a lot of variation, with the median spend being 1.3 months. Three patients, all from Rufunsa, spent 21.0, 22.5 and 23.3 months’ of the whole household’s cash income on treatment costs, and two others spent 17.0 and 18.3 months’ cash income. Eight of 64 households had to borrow from other family members or the community in order to get the financial resources for treatment of a family member with rHAT. Six of the eight recovered rHAT patients had settled their debt at the time of interviews while two had not.

### Disability adjusted life years

Baseline DALY estimates without age-weighting or discounting are presented in Table 4. The total DALY estimate for this cohort of patients, without the inclusion of long-term disabilities, was 285.2. YLLs accounted for the majority, with YLDs accounting for only 6.4%. Male patients accounted for the majority of DALYs (231.6) with female patients accounting for much less (53.6). When long-term disabilities were included in the calculation, the DALY estimate for the cohort of patients increased to 426.2, an increment of 49.5%. The proportion of YLDs also increased significantly to 37.4%, an 8.7 fold increment. When age-weighting was applied (excluding long-term disabilities), the total DALYs for the patient cohort increased from 285.2 to 336.1 (Table 5). When discounting was applied as well, this total reduced to 221.5. If the long-term disabilities were included, the total DALYs increased from 426.2 to 505.8, a total which reduced to 319.2 if discounting was applied as well.

**Table 4:** Summary of baseline disability adjusted life year calculations

Study age group	Number of deaths	Average age at death	YLLs	Number of patients	Average age at onset	Average disability weight		Average duration (years)		YLDs		DALYs	
						excluding LTDs	including LTDs	excluding LTDs	including LTDs	excluding LTDs	including LTDs	excluding LTDs	including LTDs
Males													
≤ 10	0	–	0.0	3	3.7	0.61	0.81	0.52	35.99	1.0	33.4	1.0	33.4
11–20	0	–	0.0	1	20.0	0.81	0.88	0.17	41.47	0.1	2.9	0.1	2.9
21–30	4	27.3	145.0	17	26.1	0.70	0.76	0.41	13.39	5.3	38.4	150.4	183.4
31–40	1	33.0	32.9	13	34.8	0.63	0.66	0.30	15.10	2.9	15.8	35.8	48.6
41–50	1	41.0	28.6	7	45.4	0.64	0.74	0.86	12.33	4.6	23.0	33.1	51.6
51–60	0	–	0.0	3	52.0	0.81	0.81	0.53	0.53	1.3	1.3	1.3	1.3
61–70	0	–	0.0	0	0.0	0.00	0.00	0.00	0.00	0.0	0.0	0.0	0.0
71–80	1	72.0	9.8	1	72.0	0.81	0.81	0.08	0.08	0.1	0.1	9.9	9.9
Males total	7	36.4	216.3	45	32.7	0.68	0.74	0.45	14.70	15.3	114.8	231.6	331.1
Females													
≤ 10	0	–	0	3	4.3	0.41	0.43	0.33	19.05	0.3	4.1	0.3	4.1
11–20	1	13.0	50.6	6	13.3	0.61	0.71	0.37	16.96	1.5	32.2	52.1	82.8
21–30	0	–	0	7	27.6	0.55	0.57	0.20	11.50	0.7	6.0	0.7	6.0
31–40	0	–	0	2	31.0	0.21	0.21	0.29	0.29	0.1	0.1	0.1	0.1
41–50	0	–	0	1	50.0	0.81	0.88	0.41	26.41	0.3	2.1	0.3	2.1
51–60	0	–	0	0	–							0.0	0.0
61–70	0	–	0	0	–							0.0	0.0
71–80	0	–	0	0	–							0.0	0.0
Females total	1	13.0	50.6	19	20.9	0.53	0.57	0.30	14.02	3.0	44.5	53.6	95.1
Total	8	33.5	266.9	64	29.2	0.63	0.69	0.41	14.50	18.3	159.3	285.2	426.2

Each age/sex cohort includes individual patients of different ages with different disability weights

*LTDs* long term disabilities

**Table 5:** Summary of disability adjusted life year calculations with discounting and age-weighting

	Excluding LTDs			Including LTDs		
Study age group	Age-weighted DALYs	Discounted DALYs	Age-weighted, discounted DALYs	Age-weighted DALYs	Discounted DALYs	Age-weighted, discounted DALYs
Males						
≤ 10	0.6	1.0	0.6	42.1	16.8	20.7
11–20	0.2	0.1	0.2	3.8	1.7	2.3
21–30	183.7	93.7	120.7	224.5	113.1	145.9
31–40	41.3	23.8	28.9	55.6	32.0	38.5
41–50	34.5	23.6	25.7	52.4	35.9	38.1
51–60	1.4	1.3	1.4	1.4	1.3	1.4
61–70	0.0	0.0	0.0	0.0	0.0	0.0
71–80	5.8	8.6	5.1	5.8	8.6	5.1
Males total	267.5	152.0	182.5	385.6	209.4	252.1
Females						
≤ 10	0.2	0.3	0.2	5.0	2.1	2.5
11–20	66.7	27.5	37.1	106.0	42.9	58.0
21–30	1.1	0.7	1.1	7.3	3.8	5.0
31–40	0.2	0.1	0.2	0.2	0.1	0.2
41–50	0.4	0.3	0.4	1.8	1.5	1.4
51–60	0.0	0.0	0.0	0.0	0.0	0.0
61–70	0.0	0.0	0.0	0.0	0.0	0.0
71–80	0.0	0.0	0.0	0.0	0.0	0.0
Females total	68.6	29.0	39.0	120.3	50.5	67.1
Total	336.1	181.0	221.5	505.8	259.9	319.2

*LTDs* long term disabilities

### Social consequences of human African Trypanosomiasis

The patients whose first diagnosis was not correct, or when the cause for the disease suffered was not known, they experienced stigmatization. In particular, some cases presenting with symptoms of second stage rHAT were suspected to be HIV positive, especially when the clinical signs involved mental disorder and/ or paralysis. Cognitive impairment was associated with meningitis, encephalitis and depression in these communities (common symptoms for HIV). In most cases, individuals feared to be tested for rHAT, lest they were found to be HIV positive. In a focus group discussion in Nabwalya community members stated that:

“*There are people whom we have seen who have the problem of sleeping. At times, they complain of headache. However, people fear to go to the hospital to have their blood tested, because they are afraid of being taken to have HIV/AIDs. Many would prefer staying at home and doing self-medication or seek traditional treatment than going to the hospital. When you mention to them that they are sick, they get annoyed”.*

Most rHAT cases (86%) were from remote places where belief in witchcraft was common. Focus group discussions indicated that patients who were hallucinating, and who were involved in charcoal burning, were suspected to have been bewitched (they were accused of having stolen the wood used for making charcoal). One patient was perceived to be short tempered, especially when intoxicated with alcohol. Two-thirds of school-age children (ages 8–17 years) who had recovered from rHAT (*n* = 9) dropped out of school because of lack of concentration in class. They were considered *dull witted* and as *failures* by parents and friends. rHAT cases who were being trained as Zambia Wildlife Authority Officers were stigmatised and considered to be lazy when they failed to recover after being given medication for other ailments:

“*There are so many of these situations. One of them was our wildlife scout (game ranger) student who we thought had malaria because they could not find the problem at the hospital. After undergoing malaria treatment and he was not recovering, we thought it’s a straight forward case of HIV/AIDs. He was excluded from the training and considered a failure. Later on we heard that he died and the cause of death was sleeping sickness*” (ZAWA Officers).

### Physical consequences of rHAT infection

It was estimated that 26 (40%) of the treated rHAT patients complained of pain after melarsoprol treatment. Common reported side-effects after treatment included muscle pain (89%), headache and intermittent fever (*n* = 60, 93%), sleeping disorder (*n* = 42, 65%), loss of weight and appetite (*n* = 44, 68%), nerve pain similar to the feeling of heat (*n* = 56, 88%), back pain (*n* = 57, 89%) and swelling in parts of the body (*n* = 11, 17%), especially in the hands and feet, and lymph node enlargement. These effects prevented full recovery that would allow them to do their usual work.

Most patients who recovered from second stage rHAT (*n* = 45, 70%) reported amnesia and claimed to be stigmatized. The condition was reported as worse among the school going children. One head teacher reported:

*“I don’t know what makes some of the pupils not to be able to concentrate after being absent from school for so long, they end up being worse than before. I am not aware that sleeping sickness can have such devastating consequences to the patient”* (Head Teacher).

**Fig. 3:**
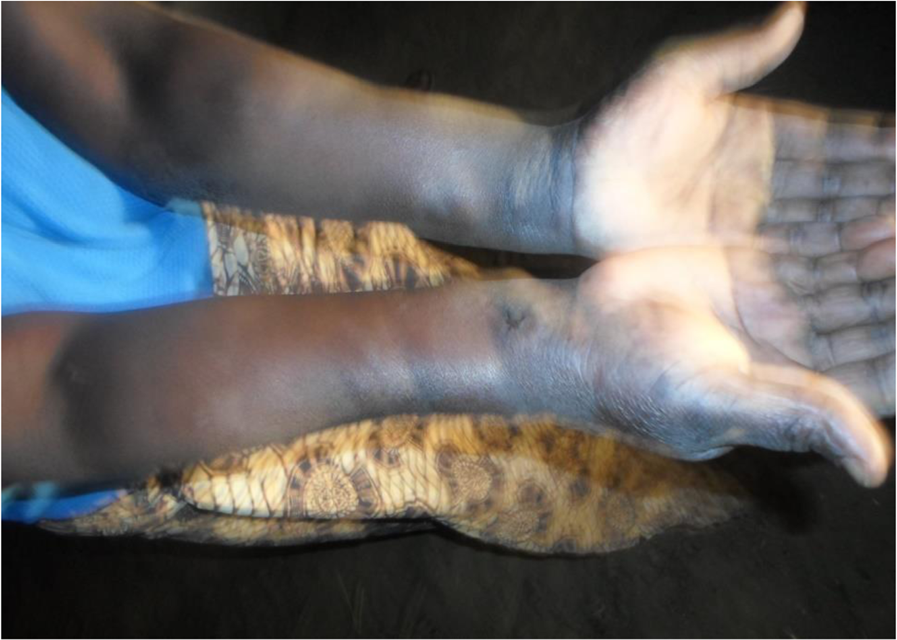
Deformed hands of a recovered rHAT patient

Physical deformities were observed in several rHAT patients (Fig. 3) which tended to become permanent disabilities. In a personal interview with a female patient who was still in hospital and had physical disabilities, she mentioned that she believed the disability was associated with melarsoprol treatment. She further mentioned that the disability resulted in her loss of self-esteem. It was also established during focus group discussions that deformities had a strong social impact on women because of loss of self-esteem as they felt that they were no longer attractive to the opposite sex.

## Discussion

This study is the first to assess the economic and social consequences of rHAT at the household level in endemic areas in Zambia. It has used an innovative approach in tracing the experience of patients from initial infection through treatment pathways and losses of income. Various short- and long-term effects on livelihood and coping strategies including physical and mental health effects on patients and affected community members were analysed.

There have been sporadic cases of rHAT reported in all the three provinces of Zambia included in this study [27, 29] where they present a significant burden on the household and the community. Patients incurred large costs relating to treatment, both direct and indirect. Of the 33 households who spent cash on treatment, the average spend was equivalent to 4.5 months of household cash income. This places a large financial burden on top of the loss of household income through ill health. Interestingly, only three out of 19 (16%) female cases spent money as against 30 out of 45 males (67%). The average spend for those patients who spent money on treatment was also less for female patients (US$ 84) compared to males (US$ 172). Costs were mostly centred on seeking treatment and hospitalisation. This money was used to purchase drugs for self-medication, for seeking treatment from traditional healers and for seeking treatment in private health care institutions. The costs described here are similar to those reported by Lutumba et al. [19].

Transport costs associated with seeking care for a rHAT patient were high. Of those patients spending money on transport to seek treatment, the average amount spent was US$ 99.3, a significant disincentive for seeking appropriate medical care. A patient would have to make several repeat visits on different occasions before being correctly diagnosed with the disease, suggesting that methods for diagnosing rHAT were ineffective. In particular, patients seeking care from traditional healers and other pharmaceutical places for diagnosis and treatment were not provided with the correct diagnosis or medication which delayed their presentation at health centres. This finding is similar to Matemba et al. [15] who reported that rHAT drugs were not readily available and evidence showed that people often sought basic health care services from more than one place.

The DALYs determined in this study were high since the majority of the rHAT cases, 45 (70%), were second stage. This observation is in line with a study in Uganda in which delays in seeking treatment and correct diagnosis increased the burden of the disease [12; 16]. However the findings in our study did not account for under-reporting as described by Fèvre et al. [16] and Matemba et al. [15], the latter finding that the high DALYs found in Tanzania’s Urambo district was due to under-reporting. The high level of second stage rHAT cases can be partly attributed to delays in health-seeking treatment and cases being initially misdiagnosed, indicative of failure to recognise the disease by health workers and/or lack of active surveillance for rHAT in affected areas [12; 13]. There was a notable difference in the number of cases identified from 2004 to 2012 when a purely passive surveillance method was used, compared with 2013–2014 when a more active surveillance method was employed. An average of three cases per year was diagnosed between 2004 and 2012, increasing to 14 in 2013 and 23 in 2014. This represents an increase of nearly eight-fold and suggests there is a high level of under-reporting. The likelihood of this being the case is reinforced by the high proportion of second stage patients [16]. It is also possible the higher incidence in 2013–2014 could be due to an outbreak, with 17 cases being identified in Mpika District, but this is considered to be less likely.

Many patients preferred to self-medicate and/or seek treatment from traditional healers until they became very ill, also contributing to late diagnoses. In agreement with Odiit et al. [12], the average period of delay for a rHAT case to attend a health care centre was two to three months and patients would be receiving treatment for a month. At the time of definitive rHAT diagnosis, the patient would have been showing symptoms of the disease for an average of 61 days and would then require hospitalization for an average of 34 days. Matemba et al. [15] reported that nearly three out of four cases of HAT were presented to health facilities in late stage of the disease in Urambo, Tanzania, and almost all of these patients, 63 (98%), were presented passively. The chances of treatment failure are higher at this stage and treatment itself can result in death. Late stage presentation has serious consequences, including a reduced chance of complete cure and increased risk of drug-associated adverse effects or disabilities. The mortality rate reported in the present study (12.5%) was higher than expected after treatment with melasoprol (3–5%), and may be an under-estimate as some patients may have died after the study concluded. This suggests that accurate and timely diagnosis, vital for treatment success, was not achieved by rural Zambian health systems during the period of study. Delayed diagnosis also raises the risk of the tsetse vectors picking up the infection when feeding on an infected person and then transmitting the disease to other people.

This study also found that HAT cases and mortality rates were higher among males than females (Table 4), with many of the affected men at the peak of their productive life [19]. The tendency of rHAT to affect productive adults is reflected in the increased DALY burden when age-weighting is applied. Adolescent men commonly become heads of household and hence get actively involved in either agricultural activities and/or hunting (poaching) in an attempt to provide food to the family. These activities predispose individuals to a higher risk of tsetse fly bites, and are mainly restricted to men. Although this explanation for an incerased risk of contracting rHAT is plausible, there are other factors which influence the detection of female cases. During the period from 2004 to 2012 when only passive case detection was used, the proportion of female cases was only 15%. In contrast, when more active case detection methods were employed in 2013 the proportion increased to 26%, and this increased further to 47% in 2014. This observation provides strong evidence that under the passive system women are less likely to be diagnosed and treated for rHAT. Combined with the observation that female patients spent much less on treatment, this suggests that this vulnerable group would benefit from targeted health care and surveillance.

An important component of this study was the evaluation of the impact of long-term disabilities on the burden of rHAT. Long-term disabilities were common in this patient cohort and are not accounted for in standard HAT DALY calculations. The impact was significant with the number of DALYs increasing by between 44% and 50%, depending on whether age-weighting or discounting was applied or not (Table 5). Being a life-long effect they are much more affected by discounting than calculations without their inclusion. In the standard DALY calculation the vast majority of DALYs result from YLL, as is expected for a disease with a high level of mortality. As a proportion of the total DALYs, the YLDs for the standard calculation were 6% of the total, and 10% of the discounted figures. However, when long-term disabilities were included the proportion of DALYs due to YLD increased to 37%. This represents an increase by a factor of more than eight (undiscounted numbers) and five (discounted numbers). There is a possibility that long-term disabilities would have resulted in premature death, but this is not included in the calculation as cases could not be followed for life and there was no basis for guessing. For this reason, assuming lifetime of disability is a ‘conservative’ estimate in the sense that it makes the DALY estimate lower rather than higher.

This study indicated that the social consequences of HAT were misunderstanding, stigma, school dropout, pain, amnesia and disability. Acquired muscular and nerve deformity had a greater effect on female cases who often lost self-esteem. Kibona [39] also found that at both community and family levels, stigmatisation, mental confusion, personality and behaviour changes (often characterized by central nervous system involvement in late-stage disease) could lead to school drop-outs, mortality, divorce and break-up of relationships, and present an unfavourable climate for bringing up children. Mental confusion/ amnesia caused the victims not to associate with friends, while pain made the victims stay home in most cases, depriving them of a social life. Stigma resulted from low levels of awareness among community members, while mental confusion may be a side effect of both late stage disease damage or a consequence of melarsoprol treatment, which is associated with high toxicity [40]. While less toxic drug regimes have been adopted for gHAT, for rHAT, melarsoprol remains the only option [40].

Mental confusion/amnesia and pain underlie the high DALY burden suffered at individual, family and community levels in the areas of study. In addition to the direct physical and mental burden it places on an individual, it also deprives them of opportunities (such as education) and results in loss of personal income and friends. This is in concordance with the findings of WHO [41] who reported that mental and behavioural disorders account for 17.6% of all YLDs in Africa. Some can last for a few weeks or months while others may last a lifetime, some are not discernible except by scrutiny while others are impossible to hide even from a casual observer.

The lack of reliable data on the occurrence and outcome of infection for rHAT together with late presentation complicates treatment and disease prognosis. Moreover, limited awareness among community members and lack of prevention programmes result in a high disease burden and DALY for rHAT in rural communities. Poor diagnostic capacity of the local health system, specific therapeutic beliefs and practices, and the reluctance of patients to spend money on treatment until the illness becomes debilitating contributed to the high number of patients seeking health care in late stages of disease. As a neglected disease, analysis of the burden of rHAT on patient and households in specific geographical foci can help stimulate resource mobilisation for prevention and control programmes at a local and national level. Assessing the social and economic impacts of a neglected disease outbreak can help to contextualise the importance of prevention both to the affected population and the costs of outbreak mitigation strategies funded by governments and donors.

## Conclusions

rHAT is a disease that emerges out of poverty both in terms of transmission dynamics and clinical outcomes, while simultaneously perpetuating and reinforcing a state of poverty among affected households. The disease impacts households on multiple levels, including in areas of income generation, agriculture and food security, children’s education and long-term health consequences such as acquired muscular, nerve deformity and mental health (amnesia). At an individual level, the disease causes loss of self-esteem due to being stigmatised resulting from the acquired long-term physical and mental disabilities. Assessing the social and economic impacts of rHAT can help to contextualise the importance of prevention both to the affected population and the costs of outbreak mitigation strategies funded by governments and donors. An evaluation of the impact of long-term disabilities should be considered in future studies on the burden of rHAT.

## Additional file

Additional file 1: Multilingual abstracts in the five official working languages of the United Nations. (PDF 657 kb)
