# Supplementary material for: Estimating the economic and social consequences for patients diagnosed with human African trypanosomiasis in Muchinga, Lusaka and Eastern Provinces of Zambia (2004–2014)
Source: Infect Dis Poverty. 2017 Oct 10;6:150. doi: 10.1186/s40249-017-0363-6 (PMC5634962; doi:10.1186/s40249-017-0363-6)
Supplement: Additional file 1: — Multilingual abstracts in the five official working languages of the United Nations. (PDF 657 kb) [file 40249_2017_363_MOESM1_ESM.pdf]

Translation of the abstract into the five official working languages of the United Nations

تقدير العواقب الاقتصادية والاجتماعية للمرضى الذين تم تشخيصهم بمرض داء المثقبيات (trypanosomiasis) الأفريقي البشري في موشينغا ولوساكا والمقاطعات الشرقية في زامبيا (2004 – 2014)

Allan Mayaba Mwiinde, Martin Simuunza, Boniface Namangala, Chitalu Miriam Chama-Chiliba, Noreen Machila, Neil Anderson, Alexandra Shaw and Susan C. Welburn

المستخلص

المقدمة: يرتبط داء المثقبيات الأفريقي الحاد (rHAT) الناجم عن تريبانوسوما بروسي روديسيانس (*Trypanosoma brucei rhodesiense*) مع ارتفاع معدل الوفيات وهو قاتل إذا ترك دون علاج. وقد درست دراسات قليلة فقط الآثار النفسية والاجتماعية والاقتصادية المترتبة على ذلك المرض (rHAT). في هذه الدراسة، تم استخدام طرق بحث نوعية وكمية مختلطة لتقييم الآثار الاجتماعية والاقتصادية ل rHAT في مقاطعات مامبوي، روفونزا، مبيكا وشاما في زامبيا.

الطريقة: تم تعقب الأفراد الذين تم تشخيصهم بمرض داء المثقبيات الأفريقي الحاد من 2004-2014 باستخدام سجلات المستشفيات والمناقشات مع المجتمعات المحلية. وقد أجريت مقابلات معهم أو مع أسرهم باستخدام استبيان منظم وأجريت مناقشات جماعية مركزة مع المجتمعات المتضررة. وقد تم التحقيق في عبء المرض باستخدام سنوات العمر المعدلة حسب الإعاقة، مع وبدون خصم ووزن العمر. وجرى أيضا التحقيق في تأثير الإعاقات الطويلة الأجل على العبء المفرط له.

النتائج: تم تحديد 64 حالة في الدراسة. وقد تم تحديد الغالبية في المرحلة الثانية، وكان معدل الوفيات مرتفعا (12.5٪). وبلغ مجموع عدد سنوات العمر المعدلة حسب الإعاقة 285 دون تخفيض أو ترجيح السن. وعند إدراج الاعاقات الطويلة الامد، ازداد هذا التقدير بنسبة 50٪ إلى 462. وزادت نسبة السنوات التي تعيش مع إعاقة من 6.4٪ إلى 37٪ من مجموع سنوات العمر المعدلة حسب العمر وغير المخصومة. وعندما طبقت طريقة رصد أكثر نشاطا في الفترة 2013-2014، ازدادت الحالات التي تم تحديدها زيادة كبيرة، مما يشير إلى وجود مستوى عال من نقص الإبلاغ. وبالمثل، زادت نسبة الإناث زيادة كبيرة، مما يشير إلى أن المراقبة السلبية قد تفشل بشكل خاص في هذه المجموعة. وفقدت مدة 4.9 شهرا من الوقت الإنتاجي لكل مريض نتيجة للعدوى. وتشمل العواقب الصحية الألم وفقدان الذاكرة والإعاقة البدنية. وشملت العواقب الاجتماعية الوصمة، والانقطاع عن التعليم، وفقدان الأصدقاء واحترام الذات. وأظهرت النتائج التي تم الحصول عليها من مناقشات المجموعات البؤرية مفاهيم خاطئة بين أفراد المجتمع المحلي والتي يمكن أن تعزى إلى نقص المعرفة حول rHAT.

الخلاصة: إن الأثر الاجتماعي والاقتصادي ل rHAT على الأسر والمجتمعات المحلية الريفية كبير. وهناك حاجة إلى تحسين المراقبة وتعزيز الخدمات الطبية المحلية من أجل التشخيص المبكر والدقيق. وينبغي إعطاء الأولوية للوقاية من الأمراض في المجتمعات المعرضة لخطر الإصابة بمرض ال rHAT ، والتدخلات التي تم وضعها لمنع انتشار الأمراض الحيوانية المنشأ من الحيوانات الأليفة والحياة البرية. وهناك حاجة إلى اتخاذ تدابير داعمة للتخفيف من الآثار الطويلة الأجل للإعاقة الناجمة عن مرض .rHAT

Translated from English version into Arabic by Khaled Zayed

评估赞比亚姆钦嘎、卢萨卡和东部省份非洲锥虫病患者的经济和社会学效应（2004-2014 年）

Allan Mayaba Mwiinde, Martin Simuunza, Boniface Namangala, Chitalu Miriam Chama-Chiliba, Noreen Machila, Neil Anderson, Alexandra Shaw and Susan C. Welburn

摘要

引言：布氏锥虫罗德西亚种（*Trypanosoma brucei rhodesiense*）引起的急性非洲锥虫病（rHAT）死亡率高，如未及时治疗则会致命。关于急性非洲锥虫病的生理、社会和经济学效应的研究极少。本文采用定性和定量研究方法来评估赞比亚曼布韦、鲁芬萨、姆皮卡和恰巴地区急性非洲锥虫病的社会经济学效应。

方法：通过医院记录和社区访谈追溯 2004-2014 年间急性非洲锥虫病患者。采用结构化问卷调查患者及其家人，并在相关社区进行焦点小组讨论。采取有或无相对值和年龄加权的伤残调整生命年（DALYs）指标来评估疾病负担。同时调查了长期残疾对该疾病负担的影响。

结果：本研究包含 64 例患者，大多数在疾病二期被确诊，病死率高（12.5%）。不包括相对

值和年龄加权的 DALYs 为 285。当包含长期残疾时，这一估值将增加 50%，达到 462。无相对值和年龄加权的 DALYs 中伤残所致生命年（YLD）的比例从 6.4%增至 37%。在 2013-2014 年采用更加主动的监测方法时，确诊病例急剧增加，表明存在许多漏报病例。同样，女性比例也大幅上升，表明被动监测对于这一群体失效
每位病人因感染平均失去了 4.9 个月的工作时间。后遗症包括疼痛、健忘和身体残疾。社会效应包括被歧视、辍学、失去朋友和自尊。焦点小组讨论的结果揭示，社区成员间的误解可能是缺乏对急性非洲锥虫病的认知导致的。
结论：急性非洲锥虫病对农村家庭和社区的社会和经济学影响巨大。需要改进监测方法并加强当地医疗服务，以便早日准确诊断疾病。对于存在急性非洲锥虫病风险的社区，应优先进行疾病预防，并采取干预措施阻断人兽共患病从家畜和野生动物到人之间的传播途径。需要采取支持性措施来减轻急性非洲锥虫病造成长期残疾的影响。

Translated from English version into Chinese by Peng Song, edited by Pin Yang

CDC CHINESE CENTER FOR DISEASE CONTROL AND PREVENTION
NATIONAL INSTITUTE OF PARASITIC DISEASES

Evaluation des conséquences économiques et sociales pour les patients diagnostiqués avec la trypanosomiase humaine Africaine à Muchinga, Musaka et les Provinces Orientales de la Zambie (2004 – 2014)

Allan Mayaba Mwiinde, Martin Simuunza, Boniface Namangala, Chitalu Miriam Chama-Chiliba, Noreen Machila, Neil Anderson, Alexandra Shaw and Susan C. Welburn

Résumé
Contexte: La forme aiguë de la trypanosomiase humaine Africaine (rTHA) causée par *Trypanosoma brucei rhodesiense* est associée à des taux élevés de mortalité, et est mortelle si elle n'est pas traitée. Seules quelques études ont examiné les impacts psychologiques, sociaux et économiques de la rTHA. Dans cette étude, des méthodes qualitatives et quantitatives de recherche ont été utilisées pour évaluer les impacts socio-économiques de la rTHA dans les districts de Mambwe, Rufunsa, Mpika et Chama da la Zambie.
Méthodes: Les personnes diagnostiquées avec la rTHA de 2004 – 2014 ont été retracées à partir des rapports des hôpitaux et des discussions avec les collectivités. Soit elles, ou leurs familles ont été interrogées à l'aide d'un questionnaire structuré et des groupes de discussion ont été menés avec les communautés concernées. Le poids de la maladie a été évalué au moyen du nombre d'années de vie corrigées de l'incapacité (AVCI), avec et sans tenir compte du vieillissement ou du gain pondéral avec l'âge. L'incidence des invalidités de longue durée liée à la rTHA a également été étudiée.
Résultats: Soixante-quatre cas ont été identifiés dans l'étude. La majorité a été identifiée en deuxième phase, et le taux de mortalité était élevé (12,5%). Le nombre total d'AVCI était de 285 sans tenir compte du vieillissement ou du gain pondéral avec l'âge. Losrque les invalidités à long terme ont été incluses, cette estimation a augmenté de 50%, soit à 462. La proportion des années vécues avec une incapacité (AVI) a augmenté de 6,4% à 37% de l'AVCI totale. Lorsque la méthode de surveillance plus active a été appliquée en 2013 – 2014, les cas identifiés ont augmenté considérablement, ce qui suggère un niveau élevé de sous-évaluation. De même, la proportion de femmes a augmenté considérablement, ce qui indique que la surveillance passive aurait être particulièrement défaillante dans ce groupe.
Un temps productif moyen de 4,9 mois a été perdu par patient par suite de l'infection. Les conséquences sur la santé incluent la douleur, l'amnésie et l'handicap physique. Les conséquences

sociales sont d’ordre de la stigmatisation, l'abandon de l'enseignement, de la perte d'amis et de l'estime de soi. Les résultats obtenus par les groupes de discussion ont révélé des malentendus parmi les membres de la collectivité, qui pourraient être attribués à un manque de connaissances sur la rTHA.

Conclusion: L'impact économique et social de la rTHA sur les collectivités et les ménages ruraux est important. Une surveillance améliorée et le renforcement des services médicaux locaux sont nécessaires pour un diagnostic précoce et précis. La prévention des maladies doit être prioritaire dans les communautés à risque à la rTHA, et des interventions doivent être mises en place afin de prévenir les maladies zoonotiques qui se propagent des animaux domestiques et sauvages. Aussi, d’autres mesures visant à atténuer les effets à long terme de l'invalidité due à la rTHA sont nécessaires.

Translated from English version into French by Ephrenm de Vera

Оценка экономических и социальных последствий для пациентов, которые были диагностированны с человеческим африканским трипаносомозом в Muchinga, Лусаке и восточных провинциях Замбии (2004 – 2014)

Allan Mayaba Mwiinde, Martin Simuunza, Boniface Namangala, Chitalu Miriam Chama-Chiliba, Noreen Machila, Neil Anderson, Alexandra Shaw and Susan C. Welburn

Аннотация

Введение: Острый человеческий Африканский трипаносомоз (rHAT), вызванный *Trypanosoma brucei rhodesiense*; таким образом связан с высокой смертностью и является смертельным, если не лечить. Лишь несколько исследований были рассмотрены психологические, социальные и экономические последствия rHAT. В этом исследовании, были смешаны качественные и количественные методы исследования, используемые для оценки социально-экономических последствий rHAT, которые положили в районах Mambwe, Rufunsa, Mpika и Chama Замбии.

Методы: Индивидуумы с диагнозом rHAT с 2004 – 2014 были прослежены с помощью больничных записей и бесед с населением. Или они сами, или члены их семей, были проведены беседы с использованием структурированной анкеты и фокус-группы проводились с затронутыми сообществами. Тяжесть заболевания была исследована с помощью жизни с поправкой на нетрудоспособности (DALYs) с и без дисконтирования и возраст-веса. Влияние долгосрочной инвалидности на груз rHAT действительно было исследовано.

Результаты: Шестьдесят четыре случая были выявлены в исследовании. Большинство было выявлено на втором этапе, и смертность была высокая (12.5%). Общее число DALYs составило 285 без дисконтирования или возраст-утяжелители. Когда долгосрочная инвалидность была включена в эту оценку, она увеличилась на 50% до 462. Доля прожитых лет с инвалидности (YLD) увеличилась с 6,4% до 37% из недисконтированных и общего числа не-возрастного-взвешенного DALYs. Когда был применен более активный метод в 2013-14, случая резко увеличивались, что указывает на высоком уровени под-отчетности. Аналогичным образом, доля женщин значительно возросла и показывает, что пассивное наблюдение может быть особенно при отсутствии этой группы.

В среднем на 4,9 месяцев продуктивного времени каждый пациент умер из-за следствия инфекции. Последствия здоровья включали боль, амнезию и физическую инвалидность. Социальные последствия включают стигмы, выпадая образование, потеря друзей и самоуважение. Результаты были полученны из фокус-групп обсуждений и выявили заблуждения среди членов сообщества, которое может быть связано с отсутствием знаний о rHAT.

Заключение: Социально-экономическое влияние о rHAT на сельских домохозяйствах общинах является существенным. Совершенствование надзора и укрепления местных медицинских услуг необходимы для ранней и точной диагностики. Профилактика заболеваний должна стать приоритетной в общинах, подверженных риску о rHAT, и мероприятия по предотвращению разлива зоонозного заболевания из домашних животных и дикой природы. Необходимо нужно вспомогательные меры для смягчения долгосрочных последствий инвалидности вследствие rHAT.

Translated from English version into Russian by Hao-Qi Zhang

Estimación de las consecuencias económicas y sociales para los pacientes diagnosticados con tripanosomiasis africana humana en Muchinga, Lusaka y las provincias orientales de Zambia (2004 - 2014)

Allan Mayaba Mwiinde, Martin Simuunza, Boniface Namangala, Chitalu Miriam Chama-Chiliba, Noreen Machila, Neil Anderson, Alexandra Shaw and Susan C. Welburn

Abstracto

Trasfondo: La tripanosomiasis africana aguda (rHAT, por sus siglas en inglés) causada por Trypanosoma brucei rhodesiense se asocia con alta mortalidad y es fatal si no se trata. Sólo unos pocos estudios han examinado los impactos psicológicos, sociales y económicos de rHAT. En este estudio se utilizaron métodos mixtos de investigación cualitativa y cuantitativa para evaluar los impactos socioeconómicos del rHAT en los distritos de Mambwe, Rufunsa, Mpika y Chama de Zambia.

Métodos: Los individuos diagnosticados con rHAT de 2004 a 2014 fueron rastreados usando registros hospitalarios y discusiones con comunidades. O ellos, o sus familias, fueron entrevistados usando un cuestionario estructurado y se realizaron discusiones de grupos focales con las comunidades afectadas. El impacto de la enfermedad se investigó utilizando años de vida ajustados por discapacidad (DALY, por sus siglas en inglés), con y sin descontar y ponderación por edad. También se investigó el impacto de las discapacidades a largo plazo a causa de rHAT.

Resultados: Sesenta y cuatro casos fueron identificados en el estudio. La mayoría se identificó en la segunda etapa, y la tasa de mortalidad fue alta (12.5%). El número total de DALYs fue de 285 sin descontar ni ponderar por edad. Cuando se incluyeron las discapacidades a largo plazo, esta estimación aumentó en un 50% a 462. La proporción de años vividos con discapacidad aumentó del 6.4% al 37% del total de DALY no descontado y no ponderado por edad. Cuando se aplicó un método de vigilancia más activo en 2013-14, los casos identificados aumentaron dramáticamente, lo que sugiere un alto nivel de sub-notificación. Del mismo modo, la proporción de mujeres aumentó sustancialmente, lo que indica que la vigilancia pasiva puede estar fracasando, especialmente en este grupo. Se perdió un promedio de 4.9 meses de tiempo productivo por paciente como consecuencia de la infección. Las consecuencias para la salud incluyeron dolor, amnesia y discapacidad física. Las consecuencias sociales incluían estigma, abandono de la educación, pérdida de amigos y autoestima. Los resultados obtenidos de las discusiones de los grupos focales revelaron conceptos erróneos entre los miembros de la comunidad que podrían atribuirse a la falta de conocimiento sobre rHAT.

Conclusiones: El impacto social y económico del rHAT en los hogares y comunidades rurales es sustancial. Se necesita una mejor vigilancia y fortalecimiento de los servicios médicos locales para un diagnóstico precoz y preciso. La prevención de enfermedades debe ser priorizada en las comunidades en riesgo de rHAT, y las intervenciones establecidas para prevenir que la enfermedad

zoonótica se derrame de los animales domésticos y la vida silvestre. Se necesitan medidas de apoyo para mitigar los efectos a largo plazo de la discapacidad debido al rHAT.

Translated from English version into Spanish by Laura C Vicente Rodriguez
